# Nucleotides Flanking the Start Codon in *hsp70* mRNAs with Very Short 5’-UTRs Greatly Affect Gene Expression in Haloarchaea

**DOI:** 10.1371/journal.pone.0138473

**Published:** 2015-09-17

**Authors:** Wenchao Chen, Guopeng Yang, Yue He, Shaoming Zhang, Haiyan Chen, Ping Shen, Xiangdong Chen, Yu-Ping Huang

**Affiliations:** Department of Microbiology, College of Life Sciences, Wuhan University, Wuhan, 430072, China; Max-Planck-Institute for Terrestrial Microbiology, GERMANY

## Abstract

Leaderless translation is prevalent in haloarchaea, with many of these leaderless transcripts possessing short 5’-untranslated regions (UTRs) less than 10 nucleotides. Whereas, little is known about the function of this very short 5’-UTR. Our previous studies determined that just four nucleotides preceded the start codon of *hsp70* mRNA in *Natrinema* sp. J7, with residues -3A and +4G, relative to the A of the ATG start codon, acting as the preferred bases around the start codon of all known haloarchaeal *hsp70* genes. Here, we examined the effects of nucleotides flanking the start codon on gene expression. The results revealed that shortening and deletion of the short 5’-UTR enhanced transcript levels; however, it led to significant reductions in overall translational efficiency. AUG was efficiently used as start codons, in both the presence and absence of short 5’-UTRs. GUG also could initiate translation, even though it was so inefficient that it would not be detected without considerably elevated transcript. Nucleotide substitutions at position -4 to +6 were shown to affect gene expression by transcript and/or translational levels. Notably, -3A and A/U nucleotides at position +4~+6 were more optimal for gene expression. Nucleotide transversions of -3A to -3C and +4G to +4T with *hsp70* promoter from either *Haloferax volcanii* DS70 or *Halobacterium salinarum* NRC-1 showed the same effects on gene expression as that of *Natrinema* sp. J7. Taken together, our results suggest that the nucleotides flanking the start codon in *hsp70* mRNAs with very short 5’-UTRs play an important role in haloarchaeal gene expression.

## Introduction

Archaea possess many features distinct from bacteria and eukaryotes. On the one hand, the archaea are prokaryotic organisms closely related to bacteria in morphology and metabolism. On the other hand, many aspects of their informational processes are more related to that of their eukaryotic homologues [1, 2]. For example, the archaeal basal transcription apparatus consists of a single eukaryotic RNA polymerase (RNAP) II-like transcriptase and two general transcription factors, TATA-element binding protein (TBP) and transcription factor B (TFB). TBP and TFB are homologues of the eukaryotic TBP and basal transcription factor TFIIB. Moreover, the archaeal promoter architecture containing the consensus sequence TATA box closely resembles that of the eukaryotic RNAP II promoters in terms of nucleotide sequence, location and function [3].

Like transcription, translation is also a key step in the gene expression process. In the process of translation, translation initiation is a primary determinant of translational efficiency. Most transcripts of protein-coding genes possess a 5’-Untranslated region (UTR) preceding the open reading frame (ORF), which are designated as leadered transcripts. Translation initiation of leadered transcripts in bacteria relies primarily on the Shine–Dalgarno (SD) sequence in the 5’-UTR, located a few nucleotides upstream of the ORF [4]. Meanwhile, the majority of eukaryotic translation initiation of leadered transcripts is driven by a ribosomal scanning mechanism that requires a “cap” at the 5’-end of the processed mRNA; the presence of a Kozak sequence (“GCCRCCAUGG”, where R represents a purine and AUG is the start codon) in the 5’-UTR helps to further enhance eukaryotic translation initiation [5, 6]. In contrast to the leadered transcript, the transcript with a 5’-UTR length less than 10 bp is categorized as leaderless. Because leaderless transcripts occur in all three domains of organisms, it has been proposed that the translation initiation acting on these transcripts might be an evolutionary oldest mechanism [7]. This hypothesis is further supported by a report that leaderless mRNAs are capable of being translated *in vitro* by bacterial, archaeal and eukaryotic translation systems [8]. However, their translation initiation mechanisms are significantly different from that of leadered mRNA. The translation of leaderless mRNA could be driven not only by 70S or 80S ribosomes, but also 30S subunits free of IF3 [9–12]. 61S ribosomes, induced by kasugamycin, lack several 30S proteins; however, they are also capable of translating leaderless mRNAs [13]. Unlike alternative codons (AUG, GUG and UUG), which can act as start codons in leadered mRNAs, initiation translation of leaderless mRNAs shows a strong dependence on AUG in *Escherichia coli* and *Haloferax volcanii* [9, 14–15].

As like bacteria, archaeal translation is directly coupled to transcription, with translation initiation occurring immediately after the beginning of mRNA synthesis [16]. However, the mechanism of translation initiation in archaea is more complex than that of bacteria. SD-dependent translation initiation, analogous to bacterial translation, is only observed in some archaeal transcripts [7, 17]. Many archaeal transcripts are led by SD free 5’-UTR. A “SD-less mechanism” has been proposed as a novel mechanism for leadered transcripts in haloarchaea, albeit its molecular details are still unknown [15, 18]. Furthermore, leaderless mRNAs are particularly prevalent in archaea, comparing with the other two domains [17, 19–20]. Bioinformatic analyses of archaeal genomes predicted that many species contain a high fraction of leaderless transcripts [18, 21].

The growing pool of experimental data and bioinformatic analyses have revealed a lot of leaderless mRNAs containing a very short 5’-UTR. Notably, 13 of 26 *Hbt*. *salinarum* leaderless transcripts, and 11 of 15 *Hfx*. *volcanii* leaderless transcripts, have also been shown to contain 5’-UTRs less than 10 nucleotides [20]. The studies of leaderless translation to date focus mostly on mRNAs lack of 5’-UTR entirely. Little is known regarding the function of these short 5’-UTRs, as well as the sequences flanking the start codon. The Hsp70 family proteins are a highly conserved group of molecular chaperones and heat shock proteins. Our previous study determined that the 5’-UTR of *hsp70* transcript from the haloarchaeon *Natrinema* sp. J7 had just 4 nucleotides [22]. In this study, to investigate the roles of nucleotides flanking the start codon of the transcript with this very short 5’-UTR on gene expression, we generated a panel of targeted deletions and point mutants. Gene expression was monitored using *β*-galactosidase gene (*bgaH*) as a reporter gene [23–24], and the effects of various combinations of native or mutated nucleotides on transcript level and translational efficiency were extensively examined in *Hfx*. *volcanii*.

## Results

### Identification of preferred nucleotides flanking the start codon in haloarchaeal *hsp70* genes

**Fig 1:**
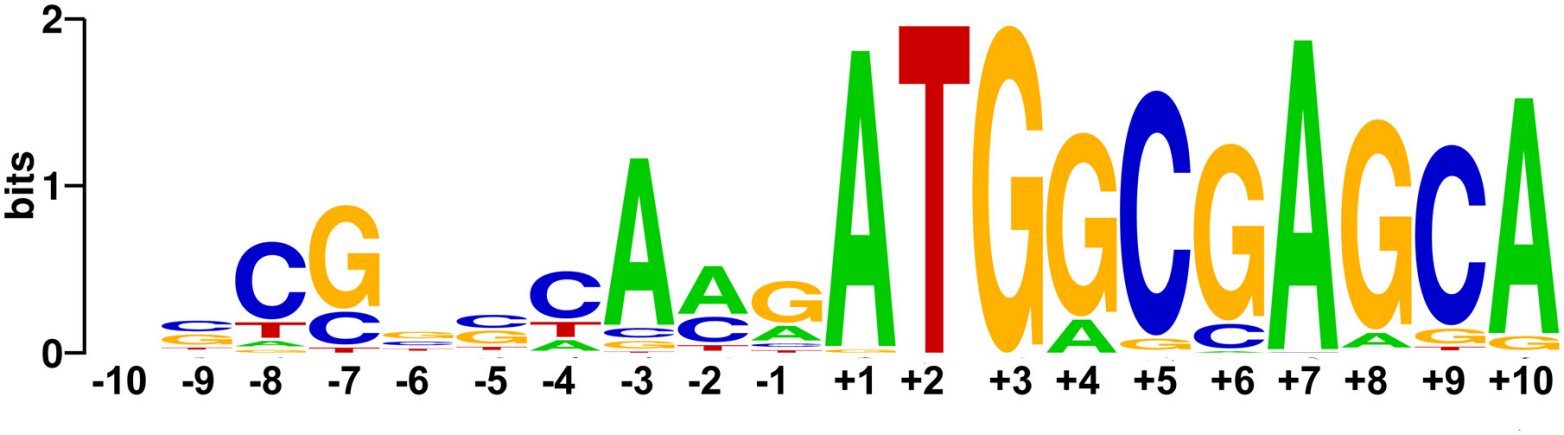
Bioinformatic analysis of the nucleotide sequences flanking the start codons of *hsp70* genes from 92 sequenced haloarchaea. The sequences from -10 to +10 were downloaded from NCBI and the sequence logo was generated by Weblogo [26]. The first base of start codon was defined as position +1.

The *hsp70* gene exists in all reported haloarchaea and is predicted to be a highly expressed gene [25]. As in *Natrinema* sp. J7, the 5’-UTR of *hsp70*, determined by RLM-RACE PCR (RNA ligase-mediated rapid amplification of cDNA ends by PCR), was also just 4 nucleotides in *Hbt*. *salinarum* NRC-1 and *Hfx*. *volcanii* DS70, which suggested that haloarchaeal *hsp70* mRNAs were likely to be with very short 5’-UTR. Further analysis of the bases flanking the start codon of *hsp70* mRNA in haloarchaea revealed a regular configuration similar to that of Kozak sequences in eukaryotes (Fig 1). Except for two strains with GUG as start codon, only AUG served as the native start codon in other sequenced haloarchaea (S1 Fig). The consensus of 4 bases upstream start codon was also very obvious, especially for strong biases of A at position -3 (the A of the AUG is +1). Because of the conservation of haloarchaeal Hsp70 sequences, the identities of the bases downstream start codon in different haloarchaea were not unexpected. However, the biases of G at position +4 caught our attention, as the +4G was very important in the Kozak sequence.

### Usage of alternative start codons on mRNA

**Fig 2:**
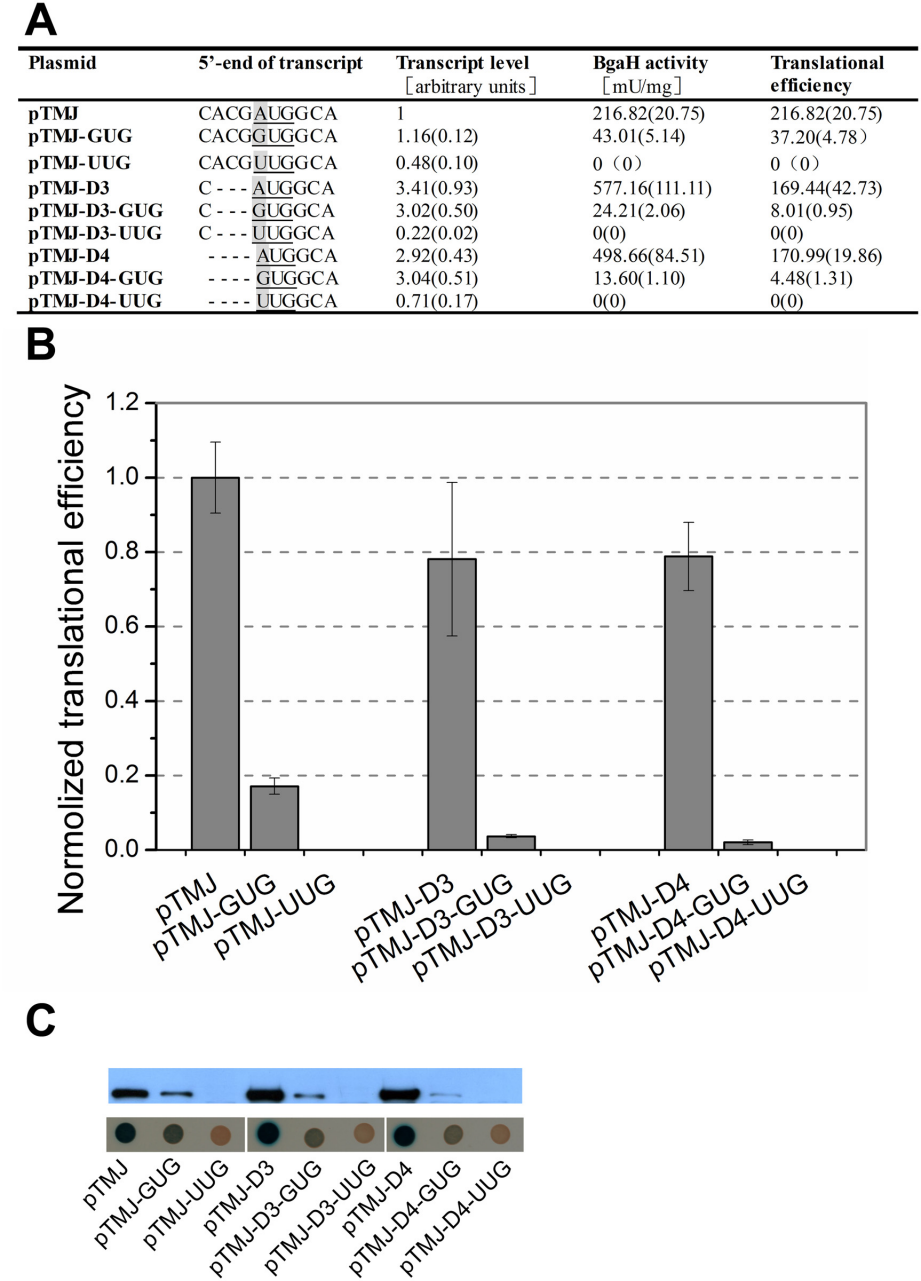
Start codon selectivities at haloarchaeal transcripts in the presence and absence of short 5’-UTR. (A) The 203-bp DNA sequence preceding the *bgaH* ORF in plasmid pTMJ was identical to the upstream sequence of *hsp70* ORF in *Natrinema* sp. J7. The bases at the 5’-end of transcripts were shown schematically. Their start codons were underlined and the mutations were in grey. The *β*-galactosidase specific activities (BgaH activities), the *bgaH* transcript levels and the translational efficiencies of *Hfx*. *volcanii* transformants were tabulated. (B) The translational efficiencies of (A) were shown schematically after normalization to that of *Hfx*. *volcanii* DS70/pTMJ. (C) The expression of the BgaH protein. Western blot analysis of the BgaH protein in total proteins was performed using anti-BgaH antibody. The *Hfx*. *volcanii* transformants were cultivated for 5 days at 45°C and then sprayed with X-Gal. The constructs present in each transformant were indicated under the colonies.

Initial experiments to examine the role of the short 5’-UTR prior to *bgaH* transcript were carried out by constructing two deletion mutants from pTMJ, one that altered the 5’-UTR sequence from 5’-CACG-3’ to 5’-C-3’ (pTMJ-D3), and the other was entirely devoid of the 5’-UTR (pTMJ-D4). In order to analyse the requirement of the start codon in translation, both in the presence and absence of the short 5’-UTR, six mutations were generated from the above three plasmids, changing the *bgaH* start codon from AUG to GUG and UUG (pTMJ-GUG, pTMJ-UUG, pTMJ-D3-GUG, pTMJ-D3-UUG, pTMJ-D4-GUG and pTMJ-D4-UUG, respectively). *Hfx*. *volcanii* strain DS70, which lacks detectable *bgaH* transcript, as well as *β*-galactosidase activity [23, 27–28], was chosen as the recipient strain and transformed with these plasmids. Then the *bgaH* mRNA and *β*-galactosidase activities in the *Hfx*. *volcanii* recombinant strains were quantified simultaneously. Meanwhile, Western blots and *Hfx*. *volcanii* transformants sprayed with X-Gal were conducted to ensure of the accuracy of enzymatic analyses (Fig 2). Here the amount of *bgaH* mRNA and *β*-galactosidase specific activity (BgaH activity) represented the *bgaH* transcript level and BgaH protein level, respectively. Translational efficiency was calculated by dividing the protein level with the transcript level. At least three independent experiments were performed in this study.

Whether with the short 5’-UTR or not, altering the start codon from AUG to GUG did not affect the abundance of *bgaH* transcript; however, the translational efficiency of *bgaH* mRNA driven by AUG start codon was found to be 5–37 times higher than that of GUG codon. In constructs in which the start codon was mutated to UUG, *bgaH* transcript levels decreased to less than half that of AUG codons, with no detectable *β*-galactosidase activity, suggesting that the UUG start codon is incapable of driving the translation of *bgaH* mRNA (Fig 2).

In constructs harbouring the AUG start codon, removing either the short 5’-UTR or ACG preceding the start codon led the amount of *bgaH* mRNA and *β*-galactosidase specific activity to increase. When the start codon was mutated to GUG, deletion of the 5’-UTR also enhanced the *bgaH* mRNA amount, but resulted in a considerable reduction of *β*-galactosidase specific activity, especially for the absence of 5’-UTR. We even doubted that the *β*-galactosidase activity could not be detected if the transcript amount was not increased so much. Whereas, using either AUG or GUG as start codon, both shortening and deletion of the 5’-UTR led the translational efficiency to reduce.

When *Hfx*. *volcanii* DS70 transformants were sprayed with X-Gal, colonies in which *bgaH* expression was driven by the AUG start codon (pTMJ, pTMJ-D3, pTMJ-D4) produced a higher degree of colour change than those containing the GUG start codon (pTMJ-GUG, pTMJ-D3-GUG, pTMJ-D4-GUG) (Fig 2C). As seen in the enzyme assay, colonies containing the UUG start codon (pTMJ-UUG, pTMJ-D3-UUG, pTMJ-D4-UUG) exhibited undetectable levels of *β*-galactosidase activity. The abundance of BgaH protein was further validated by Western blotting using a polyclonal antibody against BgaH. The results were similar to that of other assays (Fig 2C).

### The influence of nucleotide at position -3 on gene expression

Besides start codon AUG, comparative sequence alignments indicated a strong bias for A residue at position -3 upstream of *hsp70* start codon in haloarchaea (Fig 1). Kozak reported that an A or G residue at position -3 is necessary for optimal translation initiation in mammalian species [29]. To determine the effect of this preferred nucleotide on gene expression, -3A was mutated to -3G, -3C or -3T (pTMJ-3G, pTMJ-3C and pTMJ-3T, respectively).

**Fig 3:**
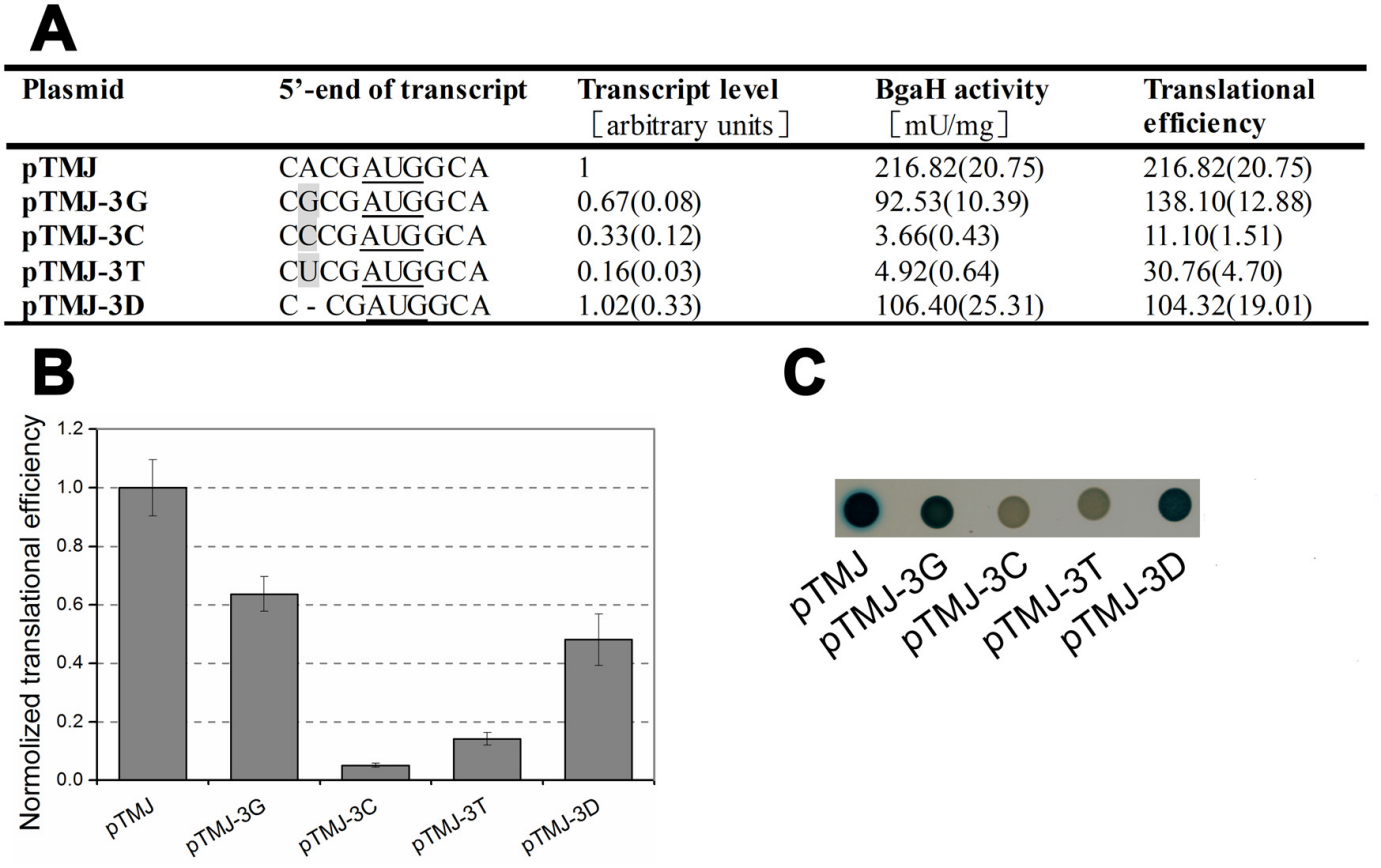
The effect of the nucleotide at position -3 on gene expression. (A) The bases at the 5’-end of transcripts were shown schematically and the mutational bases were in grey. The *β*-galactosidase specific activities (BgaH activities), the *bgaH* transcript levels and the translational efficiencies of *Hfx*. *volcanii* transformants were tabulated. (B) The translational efficiencies of (A) were shown schematically after normalization to that of *Hfx*. *volcanii* DS70/pTMJ. (C) Colonies of *Hfx*. *volcanii* transformants sprayed with X-Gal. The strains were cultivated for 5 days and then sprayed with X-Gal. The constructs present in each transformant were indicated under the colonies.

Mutation of -3A resulted in severe reductions in both the abundances of *bgaH* transcript and *β*-galactosidase specific activity. The -3G mutant retained a translational efficiency ~60% that of the -3A strain. Whereas pyrimidine residues greatly impaired gene expression, the *β*-galactosidase specific activities of the -3C and -3T mutants were nearly indistinguishable from that of *H*. *volcanii* DS70/pTM11. To further determine the role of nucleotides at position -3, an additional plasmid (pTMJ-3D) was constructed in which -3A was deleted from pTMJ, leaving only three bases in the 5’-UTR, and altering the nucleotide at position -3 from -3A to -3C. Compared with the native -3A (pTMJ), the deletion of this residue (pTMJ-3D) did not affect *bgaH* mRNA abundance; however, it did reduce translational efficiency and *β*-galactosidase specific activity to around half that of *H*. *volcanii* DS70/pTMJ (Fig 3).

### The influence of nucleotides preceding the start codon

**Fig 4:**
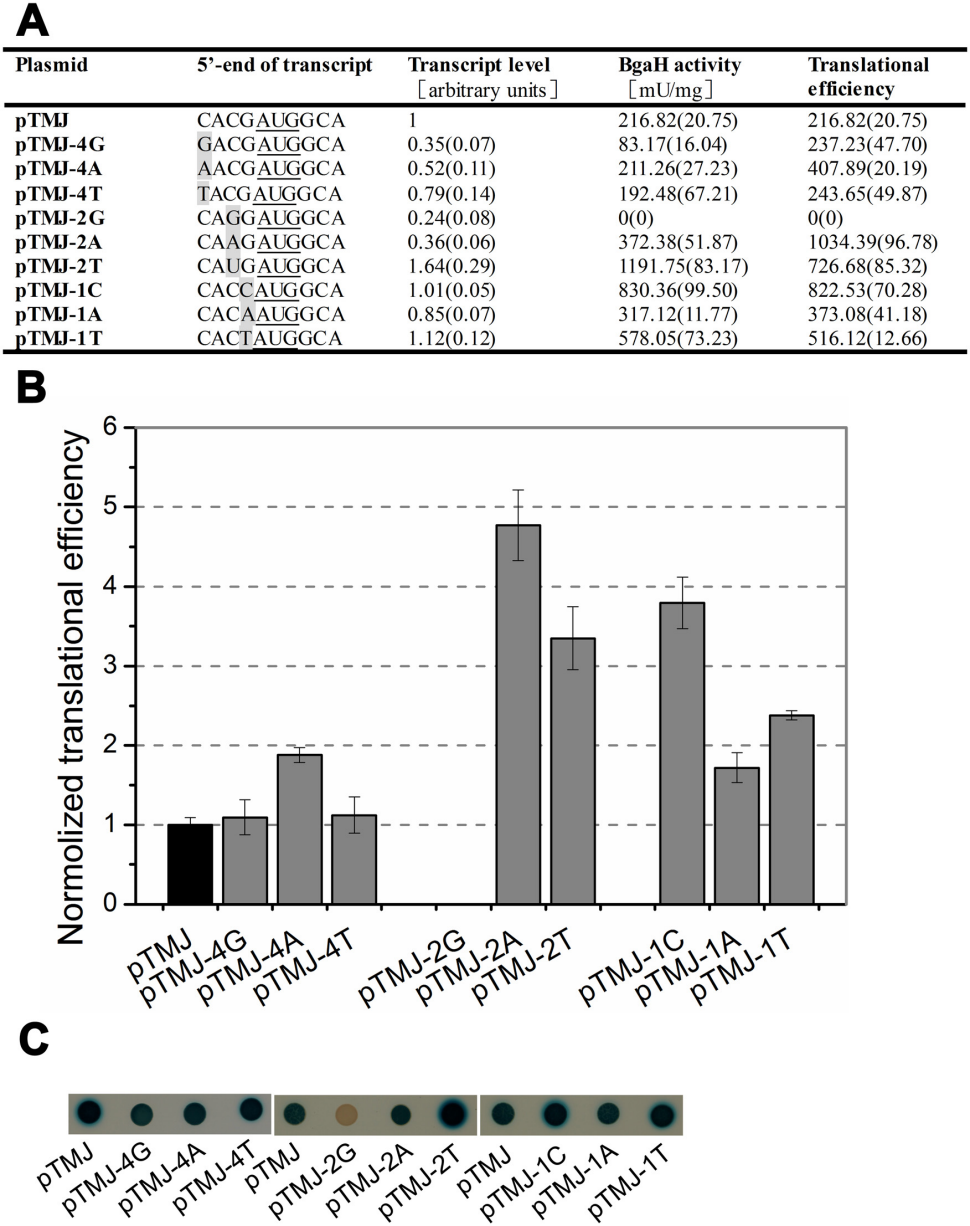
The effects of the bases in 5’-UTR other than -3A on gene expression. (A) The bases at the 5’-end of transcripts were shown schematically and the mutation bases were in grey. The *β*-galactosidase specific activities (BgaH activities), the *bgaH* transcript levels and the translational efficiencies in recombinant strains were tabulated. (B) The translational efficiencies of (A) were shown schematically after normalization to that of *Hfx*. *volcanii* DS70/pTMJ. (C) Colonies of *Hfx*. *volcanii* transformants sprayed with X-Gal. The constructs present in each transformant were indicated under the colonies.

Besides -3A, the other three bases prior to start codon also displayed biases of C/T at position -4, A/C at position -2 and G at positon -1 (Fig 1 and S1 Fig). To unravel the effects of nucleotides other than -3A on translational efficiency, mutagenesis of the other three bases preceding the start codon was made based on the plasmid pTMJ. The *β*-galactosidase specific activities, *bgaH* transcript levels and translational efficiencies of the different derivatives were summarised in Fig 4.

Nucleotide substitution at position -4 led to different degrees of reduction (35%-79%) in transcript level. Changing -4C to -4G or -4T had no effect on translational efficiency, while the translational efficiency of -4A almost increased 2-fold. The -2C substitution showed remarkable changes in both transcript level and translational efficiency. The *β*-galactosidase specific activity of -2G mutant was undetectable with a 76% reduction in *bgaH* mRNA amount. Whereas, the translational efficiencies of -2A and -2T increased more than 4-fold and 3-fold, respectively. The mutation at position -1 had no obvious impact on transcript level, but translational efficiencies of all three mutants had different degrees of increase (-1G→-1C: 379%, -1G→-1A: 172%, -1G→-1T: 238%).

### The influence of nucleotides downstream the start codon

**Fig 5:**
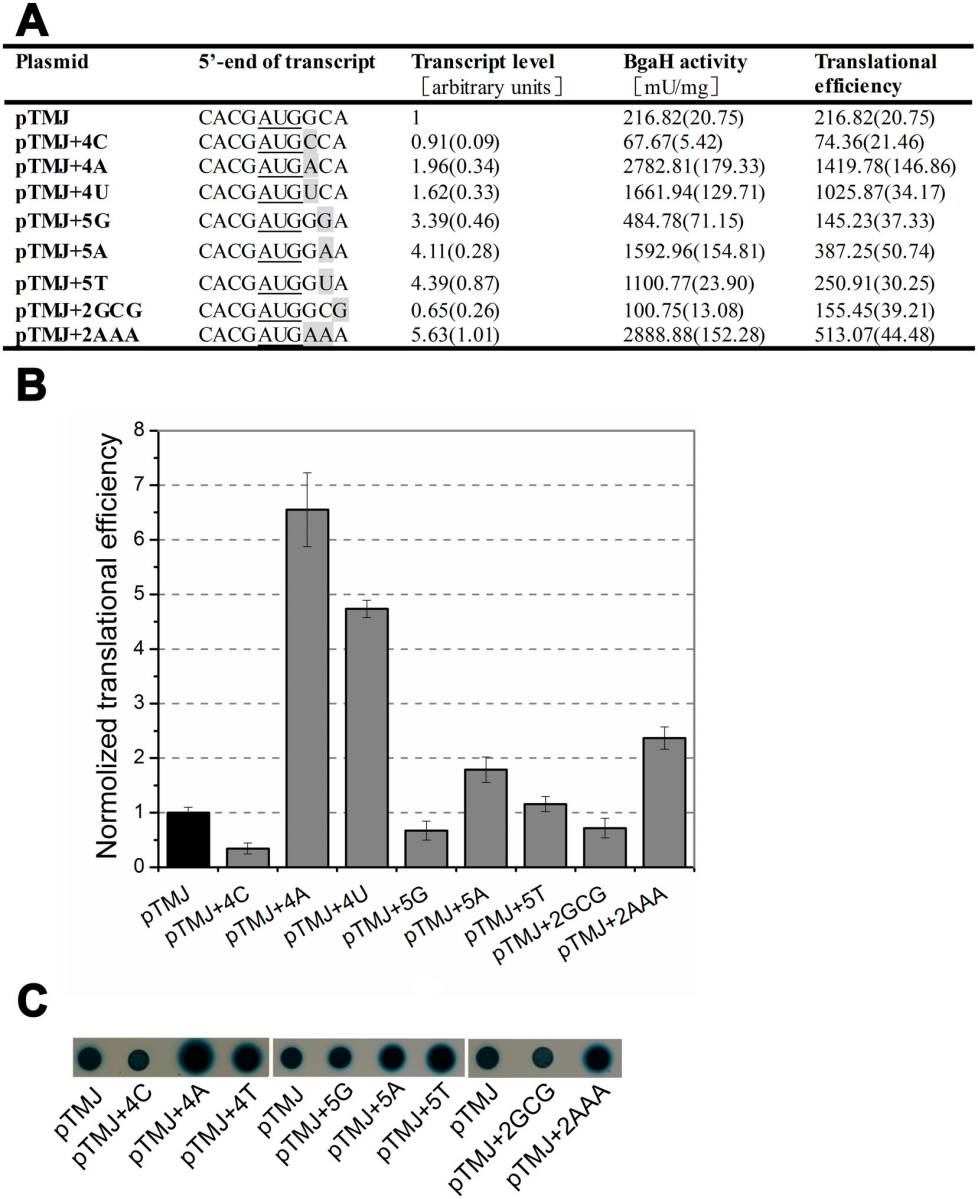
The effects of the base substitutions in the penultimate amino acid codon. (A) The bases at the 5’-end of transcripts were shown schematically and the substitutions were in grey. The *β*-galactosidase specific activities (BgaH activities), the *bgaH* transcript levels and the translational efficiencies of *Hfx*. *volcanii* strains were tabulated. (B) The translational efficiencies of (A) were shown schematically after normalization to that of *Hfx*. *volcanii* DS70/pTMJ. (C) Colonies of *Hfx*. *volcanii* transformants sprayed with X-Gal. The constructs present in each transformant were indicated under the colonies.

In order to detect whether the nucleotides downstream of the start codon affect gene expression, we attempted to alter the nucleotides at positions of +4~+6. At first, +4G was changed to +4A, +4C and +4T (pTMJ+4A, pTMJ+4C and pTMJ+4T, respectively). The results showed that mutation of +4G to +4C led the translational efficiency to reduce 2-fold. Whereas, when the +4G was changed to +4A or +4T, translational efficiency rose over 4-fold, along with relatively slight increases in transcript abundance (Fig 5). It implied the nucleotides downstream of the start codon had a significant influence on haloarchaeal gene expression. Then residue +5C was mutated to +5G, +5A or +5T (pTMJ+5G, pTMJ+5A and pTMJ+5T, respectively). As seen in Fig 5, *bgaH* transcripts and *β*-galactosidase specific activities increased obviously in all the three mutants. The translational efficiency of +5A increased while the translational efficiency of +5G decreased. Because of codon-degeneracy, altering +6A to +6G did not changed the penultimate amino acid of BgaH, but led the *bgaH* mRNA and translational efficiency to reduce about 30%. When the penultimate amino acid codon was changed from GCA to AAA, both the *bgaH* mRNA and *β*-galactosidase specific activity had a large increase, the translational efficiency also increased 2.4-fold (Fig 5).

### The influence of nucleotides at position -3 and +4 in different haloarchaeal *hsp70* genes

**Fig 6:**
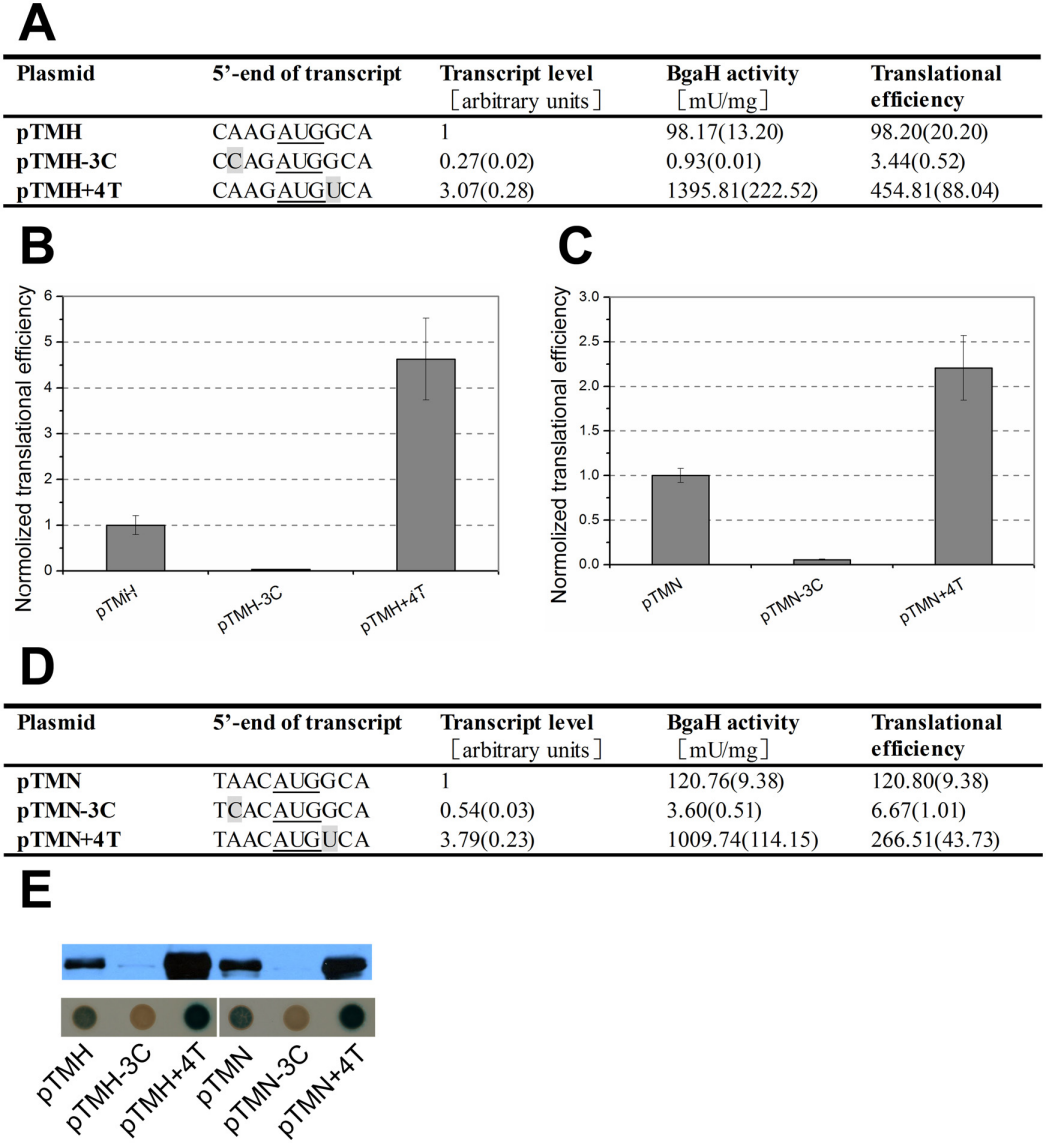
The effect of the -3C and +4T mutations on gene expression. (A) and (D) The 489-bp and 398-bp DNA sequences preceding the *bgaH* ORF in plasmids pTMH and pTMN were identical to the upstream sequences of *hsp70* ORF in *Hfx*. *volcanii* and *Hbt*. *salinarum* NRC-1, respectively. The bases at the 5’-end of transcripts were shown schematically and the mutation bases are in grey. The *β*-galactosidase specific activities (BgaH activities), the *bgaH* transcript levels and the translational efficiencies in *Hfx*. *volcanii* strains were tabulated. (B) and (C) The translational efficiencies of (A) and (D) are shown schematically after normalization to that of pTMH and pTMN, respectively. (E) The expression of the BgaH protein. Western blot analysis of the BgaH protein was carried out using anti-BgaH antibody. Colonies of *Hfx*. *volcanii* transformants were sprayed with X-Gal. The constructs present in each transformant were indicated under the colonies.

Since the nucleotides at position -3 and +4 in *hsp70* of *Hbt*. *salinarum* NRC-1 and *Hfx*. *volcanii* DS70 were also -3A and +4G, respectively. To further determine the influence of nucleotides at position -3 and +4 on gene expression, the other two *hsp70* promoters from *Hfx*. *volcanii* DS70 and *Hbt*. *salinarum* NRC-1 were cloned upstream of the *bgaH* ORF, respectively. They were named as pTM-H and pTM-N. The sequences flanking *bgaH* start codon at position -4 to +4 were mutated according to that of *hsp70* in *Hfx*. *volcanii* DS70 and *Hbt*. *salinarum* NRC-1, respectively. Then the plasmids pTMH and pTMN were obtained. Nucleotide transversions were made at position -3 and +4 using these two plasmids. The results showed that mutation of -3A to -3C in pTMH led to severe reductions in both *bgaH* transcript level and *β*-galactosidase specific actvity, including a 28-fold decrease in translational efficiency. -3C mutation upstream of *bgaH* ORF in pTMHN reduced the *bgaH* transcript level to nearly half that of native -3A, along with a nearly 20-fold reduction in translational efficiency. However, +4T substitution either in pTMH or pTMN conferred a remarkable increase in *β*-galactosidase specific activity and transcript level, even translational efficiency rose significantly (Fig 6).

## Discussion

### Use of alternative start codons

Though mammalian cells strictly employ AUG as a start codon [30–31], the usage of alternative start codons is rather common in Bacteria and Archaea [32]. For example, 82.0% of genes are initiated with an AUG, 17.8% with a GUG and 0.2% with a UUG in *Hfx*. *volcanii* [33]. All three combinations have been shown to act as alternative start codons on leadered transcripts in Bacteria and Archaea, while in haloarchaeal leaderless translation, the usage of start codon appears to be transcript-dependent. AUG was shown to act as the exclusive start codon in *Hfx*. *volcanii* leaderless transcript using the *dhfr* gene as a reporter gene [15]. However, an evidence of a GUC start codon for the initiation of leaderless mRNA translation in *Hbt*. *salinarum* showed that alternative codons are possible [34]. Our results revealed that AUG start codon was indeed more efficient than GUG start codon, regardless of the presence or absence of the very short 5’-UTR. However, GUG start codon could also drive *bgaH* mRNA translation, even though it was very weak (Fig 2).

### The effect of the nucleotides in short 5’-UTR

Nakagawa observed a disproportionate number of A/G nucleotides at position -3, relative to the initiation codon, in all 47 eukaryote species examined [35]; replacement of this residue with either a T or C nucleotide strongly impairs translation initiations in mammals [29, 36]. The preferred nucleotide at position -3 in haloarchaeal *hsp70* genes was also A residue (Fig 1 and S1 Fig). Although the -3A was essential for the optimal gene expression, deletion of this residue resulted in only a half reduction in translation efficiency (Fig 3). These data suggest that the influence of nucleotides at position -3 is context-dependent.

Besides -3A, the substitution of other bases in the 5’-UTR led the transcript level and protein level to vary with their locations (Fig 4). -4C is the original transcriptional initiation site of *hsp70* gene in *Natrinema* sp. J7. Although altering the -4C to other bases reduced the amount of *bgaH* mRNA, it did not have obvious influence on translational efficiency. Whereas, changing the -1G to other bases had no obvious influence on the *bgaH* transcript level, but led the translational efficiency to increase. It seemed that the nucleotides at position -4 just affected transcription, and that the nucleotides at position -1 only influenced translation. The effects of base substitutions at residue -2 were complex. -2A/T may be the optimal nucleotide for *hsp70* gene expression in haloarchaea. It was interesting to find that -2G mutant had no detectable *β*-galactosidase activity. Maybe this is the reason why there was almost no G located at position -2 in haloarchaeal *hsp70* (S1 Fig).

### The effect of the nucleotides downstream the start codon

Earlier studies indicated that A/U nucleotides downstream of the start codon could increase translation initiation in *E*. *coli* [37–38]. Our results of altering the nucleotides at positions of +4~+6 also showed that mutations of C/G to A/U would lead to increase in translational efficiencies in haloarchaea. Notably, when the N-terminal penultimate amino acid codon was altered from GCA to AAA, the *β*-galactosidase specific activity reached the largest with the large increase of *bgaH* transcript (Fig 5). Among the nucleotides at positions of +4~+6, the nucleotide at position +4 had a strong influence on the translational efficiency. When nucleotide A or U was located at position +4, the penultimate amino acid codon was mutated to either a threonine codon (ACA) or serine codon (UCA) and resulted in the translational efficiency to rise dramatically. Alternatively, the translational efficiency of the proline codon (CCA) was just one-third that of the control alanine codon (GCA). Compared with the nucleotide at position +4, the nucleotides at position +5 and +6 seemed to have a slight influence on the translational efficiency. It seemed that the importance of +4 residue on translation was related to its location adjacent to the start codon AUG.

The N-terminal penultimate amino acid of BgaH was altered due to the mutations of +4G and +5C to other nucleotides in this study. Post-translation modification is common and able to affect the stability of proteins in haloarchaea [39–40]. Amino acid substitutions of the penultimate glutamine for small and uncharged amino acids in α1 protein influence its post-translation modification, and then lead the protein levels to alter [41]. Thus, the observed BgaH amounts in Fig 5A might be affected by protein stabilities. So far, the information about post-translation modification of BgaH protein is only the removal of the initiating methionine, which is accomplished by methionine aminopeptidases (MAPs). Comparison analyses suggest that the cleavage efficiency of MAPs is conserved in three domains and affected by the penultimate residue [42]. If the penultimate residue has a small radius of gyration, the N-terminal methionine residue can be removed more efficiently. For *Hfx*. *volcanii*, MAPs generally cleave nascent proteins when the penultimate residues are small and uncharged amino acids (Glycine, Alanine, Proline, Valine, Serine or Threonine) [39]. In the +4G swap experiment, the penultimate alanine of BgaH and its substitutions (proline, threonine and serine) were small and uncharged amino acids. In addition, the cleavage efficiency of N-terminal methionine is high (nearly 90%-100%) if these four amino acids are in the second position [42]. This should minimize the influence of the penultimate amino acid substitution on the protein stability to a considerable extent in *Hfx*. *volcanii*. Therefore, we deemed that the differences of the observed BgaH amounts could reflect the protein levels in *Hfx*. *volcanii*. However, much more experimentation is necessary to confirm whether the penultimate amino acid affect the stability of BgaH protein in *Hfx*. *volcanii*.

### The 5’-UTR and gene expression

Using either AUG or GUG as a start codon, shortening or deletion of the 5’-UTR reduced the translational efficiencies but increased the transcript levels. When GUG was used as start codon, deletion of the very short 5’-UTR led the translational efficiency to decrease sharply (Fig 2). It suggests that the presence of the very short 5’-UTR is more important for GUG to drive translation. Leaderless transcripts containing several nucleotides upstream of the start codon are common in archaea, as well as some primitive eukaryotes [20, 43]. Our results display that the very short 5’-UTR is able to affect the gene expression. The appropriate expression is essential for some genes, especially for *hsp70*, whose overexpression might cause a defect of the Hsp70 machine and be harmful to cell cycle and survival [44–45]. Therefore, it implies that these very short 5’-UTRs are beneficial for organisms to live, especially in a nutrient limited environment.

### Phylogenetic analysis of all reported haloarchaeal strains

Hsp70 as a highly conserved molecular chaperone, the regulation of its gene has been extensively characterised in Bacteria and Eukarya. Although Hsp70 is present in all reported haloarchaea. However, whether conserved elements in the haloarchaeal *hsp70* promoters exist, or what role Hsp70 might play in haloarchaea, is unknown [46]. Bioinformatic analysis of the nucleotides flanking the start codon of haloarchaeal *hsp70* genes showed that the preferred nucleotides at position -3 and +4 were A and G, respectively. Different nucleotides at position -3 or +4 were observed in 15 of 92 strains included in this study (S1 Fig).

**Fig 7:**
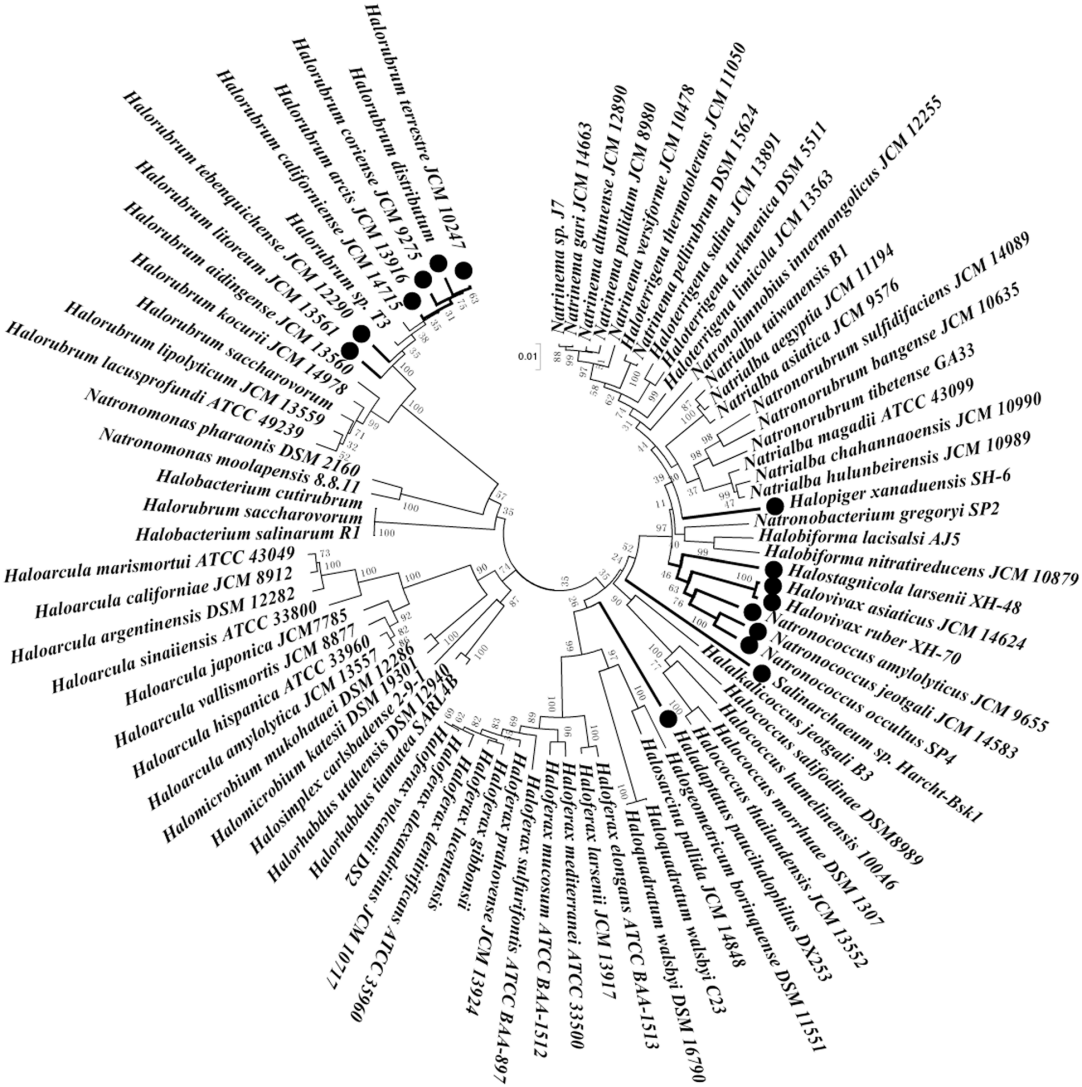
Phylogenetic neighbor-joining trees of the haloarchaeal 16S rDNA sequences. The nucleotide sequences flanking the initiation codons of their corresponding *hsp70* genes had been shown in S1 Fig. The strains, in which nucleotides flanking the initiation codons of *hsp70* gene were not in consensus, were marked with thick lines and ●.

The nucleotide at position -3 is G in *Halopiger xanaduensis* SH-6. Both *Haladaptatus paucihalophilus* DX253 and *Salinarchaeum* sp. Harcht-Bskl have G residue at position +4. Twelve strains harbour different nucleotides at position -3 and +4; two of them use GUG as start codon. To examine the evolutionary relationship between haloarchaeal strains containing *hsp70* genes, species were aligned based upon their 16S rDNA sequences using CLUSTALW and visualised using Molecular Evolutionary Genetics Analysis software (MEGA version 5.05; Arizona State University, Tempe, AZ) [47]. Strains with different nucleotides at position -3 and/or +4 of *hsp70* genes are concentrated within two groups in the phylogenetic trees (Fig 7). This means that the nucleotide sequences flanking the start codon, each with its own unique function, might evolve over time.

Why some mRNAs contain very short 5’-UTRs of less than 10 nucleotides remains unknown. Here, our studies demonstrate that the nucleotides, flanking the start codons in *hsp70* mRNAs with very short 5’-UTRs, play an important role in both transcription and translation. Future studies regarding its effect under specific physiological settings, and the biological significance of these effects on transcription and translation, will provide valuable insights into the mechanisms of translation initiation and the evolution of this process.

## Materials and Methods

### Micro-organisms, media and growth conditions

*Hfx*. *volcanii* DS70 and *Hbt*. *salinarum* NRC-1 were kindly provided by Professors Thorsten Allers and Shiladitya DasSarma, respectively. The *Natrinema* sp. J7 strain was grown in 20% (salt, w/v) modified growth medium (MGM) at 45°C [22], while *Hfx*. *volcanii* and *Hbt*. *salinarum* NRC-1 were cultivated aerobically in 18% (salt, w/v) MGM at 45°C. Cultures were supplemented with 0.3 μg ml^-1^ novobiocin to select for resident plasmids. *E*. *coli* strains DH5α and JM110 were used as hosts for cloning vectors. The strains were grown in Luria–Bertani medium at 37°C, and ampicillin was added at 100 μg ml^-1^ when appropriate.

### Bioinformatic analysis of the nucleotide sequences flanking the start codon

Sequenced haloarchaeal *hsp70* genes were searched and downloaded from NCBI. So far, 92 *hsp70* sequences were collected in total. As the first base of start codon was defined as position +1, the sequences from -10 to +10 of all *hsp70* genes were retrieved and aligned to obtain the sequence logo of the flank region of translation start codon using Weblogo 3, version 2.8.2 [26].

### Plasmid construction

All plasmids used in this study and their characteristic features are summarised in S1 Table. The oligonucleotides used for plasmid construction are listed in S2 Table.

The *hsp*70 promoter of *Hbt*. *salinarum* NRC-1 was amplified using PCR primers NRC-pro-f and NRC-pro-r, each of which contained two restriction sites: *Nsi*I and *Bgl*II in the upstream primer, and *Nco*I and *Not*I in the downstream primer. The PCR product then was cloned into shuttle vector pTA230 using *Nsi*I and *Not*I enzymes; the resulting plasmid was digested with *Ssp*I*/Bam*HI and then ligated with the *Sma*I*/Bam*HI-digested product of plasmid pMLH32, which contained the *gyrB* gene. The resulting plasmid was designated pTM11. Using pMLH32 as a template, the *bgaH* gene was amplified using primers *bgaH*-f and *bgaH*-r, and was inserted into pTM11 using *Nco*I and *Bam*HI. The resulting plasmid was named pTM-N. Next, the plasmid was digested with *Bgl*II and *Nco*I, and the *hsp*70 promoter of pTM-N was replaced with the *hsp*70 promoter fragment of *Hfx*. *volcanii* DS70 and *Natrinema* sp. J7 in plasmids pTM-H and pTM-J, respectively.

pTMJ was constructed using plasmid pTM-J as a template. To maintain the same upstream sequence and +4G as that of *Natrinema* sp. J7 *hsp*70, the *Nco*I site (CCATGG) of pTM-J was changed to CGATGG in pTMJ. Using two pairs of primers, two different PCR fragments were generated, which comprised the promoter of *hsp*70, the 5’-terminal sequence of *hsp70* transcripts (-4~+4) and a partial *bgaH* gene, respectively. These two PCR fragments were purified and fused into one fragment via overlapping PCR using primers J7 pro-f and Kozak-R2. The resulting PCR fragment was purified, digested with *Bgl*II and *Kpn*I and used to replace an equivalent length fragment in pTM-J; the resulting plasmid was named pTMJ. Similarly, plasmids pTMH and pTMN were constructed based on plasmids pTM-H and pTM-N, respectively. The *Nco*I site (CCATGG) of the corresponding plasmids was changed to AGATGG in pTMH and ACATGG in pTMN, respectively. Using the same method, a series of plasmids carrying the point mutation and deletion mutation were constructed based on the corresponding plasmids.

### Determination of *bgaH* transcript levels

Total RNA was isolated from log-phase cultures (OD_600_ = 1.0–1.5) using the TRIzol Max Bacterial RNA Isolation Kit (Invitrogen, Carlsbad, CA), following the manufacturer’s instructions. DNase treatment and reverse transcription were performed using the PrimeScript RT reagent kit with gDNA eraser (Perfect Real Time; TaKaRa, Dalian, China). The reaction mixture contained less than 1 μg RNA in a final volume of 20 μl. Real-time PCR was carried out using TaKaRa SYBR Premix Ex Taq (TaKaRa); quantitative PCR samples contained 2.0 μl diluted cDNA, and 10 μM each of forward and reverse primers in a final volume of 20 μl. Reactions were conducted using a StepOnePlus Real-Time PCR system (Applied Biosystems, Foster City, CA) under the following conditions: 95°C for 30 s, followed by 40 cycles at 95°C for 5 s and 60°C for 30 s. Each reaction was done in triplicate. To detect any possible DNA contamination, cDNA was replaced with DNase-treated RNA.

16S rRNA transcript levels were used as an internal control using primer pair 16S rRNA–RT-f and 16S rRNA–RT-r. Real-time PCR results were analysed using the ^ΔΔ^Ct method [48]. The Ct levels of the control transcripts 16S rRNA were used to normalise Ct levels of the *bgaH* transcripts. The *bgaH* level of the chromosomal gene copy was determined by *Hfx*. *volcanii* DS70/ pTM11.

### *β*-galactosidase assay and translational efficiency analysis

3 μl culture of *Hfx*. *volcanii* transformants (OD_600_ = 1.0) were dropped on 18% MGM solid medium and cultivated for 5 days at 45°C. A solution of X-Gal in dimethyl formamide (10 mg ml^-1^) was then sprayed on colonies using a hand-atomiser (perfume spray bottle) and the plates were incubated at room temperature for 2 h [49].

*β*-Galactosidase specific activity in cell lysates was measured using the ONPG assay, as described by Holmes *et al*. [50]. The protein concentration was determined by a Bradford assay using bovine serum albumin (BSA) as a standard. Translational efficiencies were calculated by dividing the *β*-galactosidase specific activities with the transcript levels. At least three independent experiments for all analyses were performed, and average values and standard deviations were calculated.

### Western blot analysis

Cells were grown at 45°C until the mid-logarithmic growth phase; then the protein concentration was determined by the Bradford assay method. Western blot analysis was performed using 2 μg total protein, resolved on a 10% acrylamide gel and transferred to a nitrocellulose membrane using a Transblot cell (Bio-Rad, Hercules, CA). The membrane was then incubated with polyclonal anti-BgaH rabbit antiserum (1:1000 dilution). Horseradish peroxidase-conjugated goat anti-rabbit IgG (Millipore, Billerica, MA) was used as a secondary antibody (1:10000 dilution). Immunoblots were developed with SuperSignal West Pico Substrate (Pierce, Rockford, IL).

### Phylogenetic analysis of haloarchaeal 16S rDNA

The 16S rDNA sequences of all reported haloarchaea (DQ874620, AB663462, AB663460, AB663463, AB663465, AB663449, NR 102444, AB663448, NR 074238, AB663445, AB663472, NR 029124, AB663454, AB663455, AB663476, AB663474, NR 028147, NR 074183, AB663456, AB663457, NR 102918, NR 102442, NR 028201, AB663436, NR 028169, AB663452, NR 102445, AB663468, AB663469, NR 102453, NR 103951, NR 102920, NR 040777, NR 043474, NR 043387, AB663372, NR 043744, NR 102892, AB663429, HM165235, NR 074200, NR 043987, AB663377, NR 028212, NR 043988, NR 028204, AB663381, D13378, AB081732, NR 028215, AB663373, NR 074218, NR 044108, NR 074206, AJ586107, JQ346761, NR 044337, AB605776, NR 102900, AB663358, EF645684, D14130, NR 044335, AB477984, NR 074201, NR 074204, AB663363, AH000908, NR 102519, NR 074179, NR 074194, AB663415, X82167, AB663413, AB663402, AB663416, AB663421, JQ936845, AB663406, AB663405, AB663409, D63572, AB663422) were downloaded from NCBI and aligned using the CLUSTALW multiple sequence alignment programme for Windows XP. Phylogenetic analysis was conducted using Molecular Evolutionary Genetics Analysis software (MEGA version 5.05) [47]. Trees were constructed by neighbour-joining using the p-distance method. Bootstrap values were calculated based on 1000 computer-generated trees.

## Supporting Information

S1 Fig The nucleotide arrays from position -10 to +10 of sequenced haloarchaeal *hsp70* genes. The first base of translation start codon was defined as position +1. The consensus nucleotides were indicated in grey, the inconformities were shown in red letters. The list arranged in alphabetical order according to the names of the strains.
(TIF)

S1 Table Plasmids used in this study and their characteristic features. (DOC)

S2 Table Primers used for plasmid construction and Realtime PCR. (DOC)
