# Supplementary figures and images for: Nucleotides Flanking the Start Codon in hsp70 mRNAs with Very Short 5’-UTRs Greatly Affect Gene Expression in Haloarchaea
Source: PLoS One. 2015 Sep 17;10(9):e0138473. doi: 10.1371/journal.pone.0138473 (PMC4574771; doi:10.1371/journal.pone.0138473)

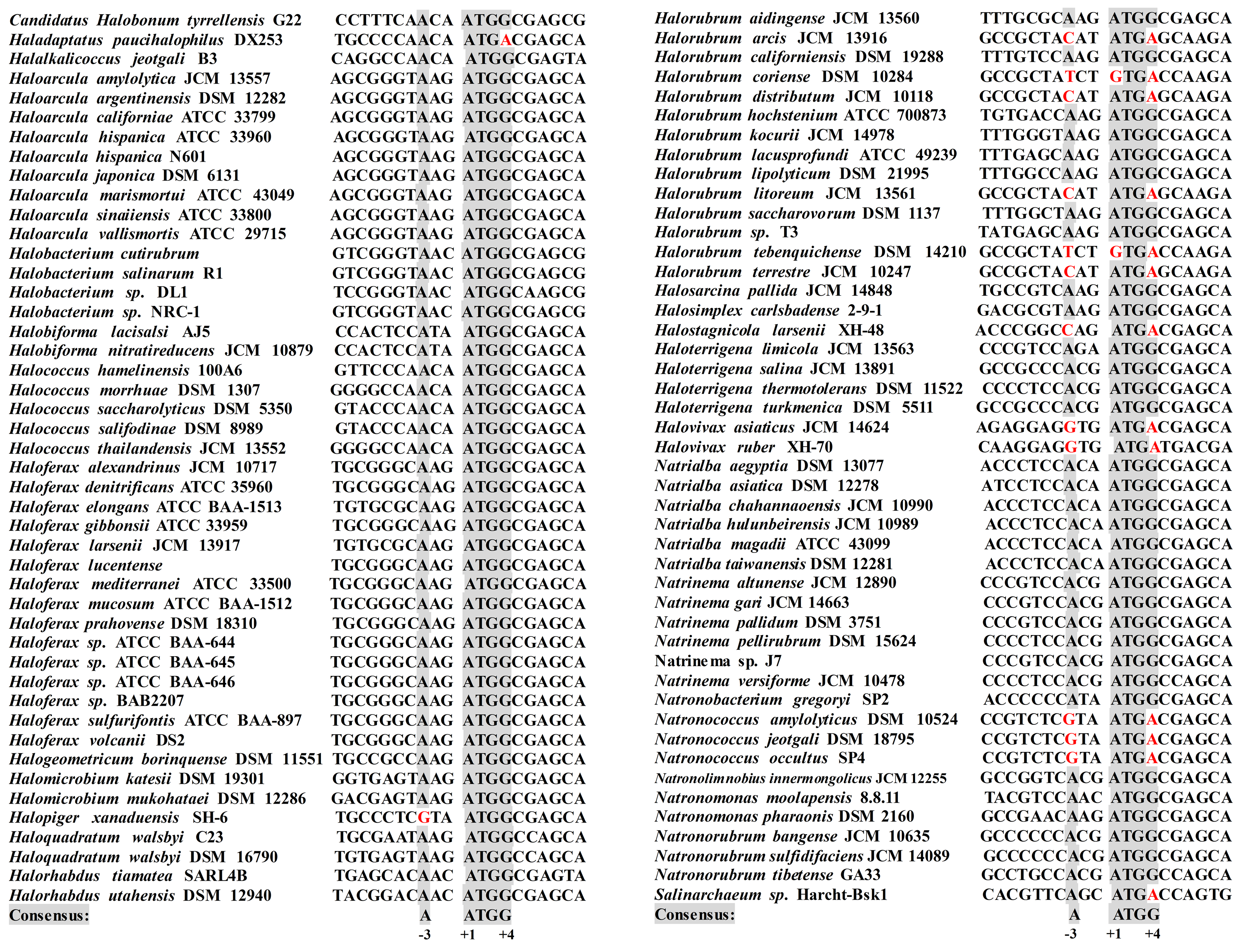

Supplement: S1 Fig — The first base of translation start codon was defined as position +1. The consensus nucleotides were indicated in grey, the inconformities were shown in red letters. The list arranged in alphabetical order according to the names of the strains. (TIF) [file pone.0138473.s001.tif]
