# Supplementary material for: Nucleotides Flanking the Start Codon in hsp70 mRNAs with Very Short 5’-UTRs Greatly Affect Gene Expression in Haloarchaea
Source: PLoS One. 2015 Sep 17;10(9):e0138473. doi: 10.1371/journal.pone.0138473 (PMC4574771; doi:10.1371/journal.pone.0138473)
Supplement: S1 Table — (DOC) [file pone.0138473.s002.doc]

| S1 Table. Plasmids used in this study and their characteristic features. | | | |
| --- | --- | --- | --- |
| **Plasmid** | **Feature** | **Parental plasmid** | **Reference** |
| pMLH32 | shuttle vector for *E. coli* and *Hfx. volcanii* with *bgaH* and Nov^R^ | **-** | Holmes and Dyall-Smith, 2000 |
| pTA230 | shuttle vector for *E. coli* and *Hfx. volcanii* with pHV2 replication origin | **-** | Allers *et al*., 2004 |
| pTM1 | pTA230 with insertion of *Nsi*I-*Not*I PCR fragment containing *hsp70* promoter of *Hbt. salinarum* NRC-1 | pTA230 | This study |
| pTM11 | pTM1 with insertion of *Sma*I-*Bam*HI *gryB* fragment (Nov^R^) from pMLH32 | pTM1 | This study |
| pTM-N | *bgaH* gene under the control of 398-bp *hsp70* promoter from *Hbt. salinarum* NRC-1 | pTM11 | This study |
| pTM-H | *bgaH* gene under the control of 489-bp *hsp70* promoter of *Hfx. volcanii* | pTM-N | This study |
| pTM-J | *bgaH* gene under the control of 203-bp *hsp70* promoter from *Natrinema sp.* J7 | pTM-N | This study |
| pTMJ | elimination of the *Nco*I site upstream of *bgaH* gene and addition of the native 5’-UTR of *hsp70* from *Natrinema sp.* J7 | pTM-J | This study |
| pTMJ-5A | substitution of -5C to -5A | pTMJ | This study |
| pTMJ-4A | substitution of -4C to -4A | pTMJ | This study |
| pTMJ-3G | substitution of -3A to -3G | pTMJ | This study |
| pTMJ-3C | substitution of -3A to -3C | pTMJ | This study |
| pTMJ-3T | substitution of -3A to -3T | pTMJ | This study |
| pTMJ-2A | substitution of -2C to -2A | pTMJ | This study |
| pTMJ-1T | substitution of -1G to -1T | pTMJ | This study |
| pTMJ-GUG | GUG as start codon of *bgaH* gene with a native 4nt 5’-UTR of *hsp70* | pTMJ | This study |
| pTMJ-UUG | UUG as start codon of *bgaH* gene with a native 4nt 5’-UTR of *hsp70* | pTMJ | This study |
| pTMJ+4C | substitution of +4G to +4C | pTMJ | This study |
| pTMJ+4A | substitution of +4G to +4A | pTMJ | This study |
| pTMJ+4T | substitution of +4G to +4T | pTMJ | This study |
| pTMJ-D3 | shortening 5’-UTR of *hsp70* to one base | pTMJ | This study |
| pTMJ-D3-GUG | GUG as *bgaH* start codon with 5’-UTR of one base | pTMJ-D3 | This study |
| pTMJ-D3-UUG | UUG as start codon of *bgaH* gene with 5’-UTR of one base | pTMJ-D3 | This study |
| pTMJ-D4 | deletion of the native 5’-UTR of *hsp70* | pTMJ | This study |
| pTMJ-D4-GUG | GUG as *bgaH* start codon lack of 5’-UTR | pTMJ-D4 | This study |
| pTMJ-D4-UUG | UUG as start codon of *bgaH* gene lack of 5’-UTR | pTMJ-D4 | This study |
| pTMH | elimination of the *Nco*I site upstream of *bga*H gene and addition of the native 5’-UTR of *hsp70* from *Hfx. volcanii* to *bgaH* | pTM-H | This study |
| pTMH-3C | substitution of -3A to -3C | pTMH | This study |
| pTMH+4T | substitution of +4G to +4T | pTMH | This study |
| pTMN | elimination of the *Nco*I site upstream of *bga*H gene and addition of the native 5’-UTR of *hsp70* from *Hbt. salinarum* NRC-1 to *bgaH* | pTM-N | This study |
| pTMN-3C | substitution of -3A to -3C | pTMN | This study |
| pTMN+4T | substitution of +4G to +4T | pTMN | This study |
