# Supplementary material for: Nucleotides Flanking the Start Codon in hsp70 mRNAs with Very Short 5’-UTRs Greatly Affect Gene Expression in Haloarchaea
Source: PLoS One. 2015 Sep 17;10(9):e0138473. doi: 10.1371/journal.pone.0138473 (PMC4574771; doi:10.1371/journal.pone.0138473)
Supplement: S2 Table — (DOC) [file pone.0138473.s003.doc]

**S2 Table. Primers used for plasmid construction and Realtime PCR.**

| **Name** | **Sequence(5’-3’)** | **Application** | **Plasmid** |
| --- | --- | --- | --- |
| *bgaH*-f | TGAGCGGCCGCCATGGCAGTTGGTGTCTGCTATTTCC | amplification of *bgaH* ORF | pTM-N |
| *bgaH*-r | AGTTGGGATCCGCTTCCGCGTGTGTACACGCGAGGA | amplification of *bgaH* ORF | pTM-N |
| NRC-pro-f | GCGCAATGCATAGATCTGGCGTTCAGGCCGGACAGCA | amplification of *hsp70* promoter region of *Hbt. salinarum* NRC-1 | pTM1 |
| NRC-pro-r | TGCGCGGCCGCCATGGTACCCGACAGTACGGAGCGTTTTC | amplification of *hsp70* promoter region of *Hbt. salinarum* NRC-1 | pTM1 |
| J7 pro-f | TTCAGATCTCGAAGCGGACGAGTAAC | amplification of *hsp70* promoter region of *Natrinema* sp. J7 | pTM-J |
| J7 pro-r | TCGCCATGGTGGACGGGTATTGT | amplification of *hsp70* promoter region of *Natrinema* sp. J7 | pTM-J |
| HV pro-f | GTGAGATCTTCGCTGCCGCCGTTCC | amplification of *hsp70* promoter region of *Hfx. volcanii* | pTM-H |
| HV pro-r | TCTCCATGGTGCCCGCAAATACCGTCTT | amplification of *hsp70* promoter region of *Hfx. volcanii* | pTM-H |
| R-ACG-J | GACACCAACTGCCATCGTGGACGGGTATT | site directed mutagenesis with pTM-J as template | pTMJ |
| ACG-5A-J | CAACTGCCATCGTGTACGGGTATTGTGCG | site directed mutagenesis with pTMJ as template | pTMJ-5A |
| ACG-4A-J | ACCAACTGCCATCGTTGACGGGTATTGTGCG | site directed mutagenesis with pTMJ as template | pTMJ-4A |
| ACG-3C-J | CACCAACTGCCATCGGGGACGGGTATTGTGC | site directed mutagenesis with pTMJ as template | pTMJ-3C |
| ACG-3G-J | CACCAACTGCCATCGCGGACGGGTATTGTGC | site directed mutagenesis with pTMJ as template | pTMJ-3G |
| ACG-3T-J | CACCAACTGCCATCGAGGACGGGTATTGTGC | site directed mutagenesis with pTMJ as template | pTMJ-3T |
| ACG-3D-J | CACCAACTGCCATCGGGACGGGTATTGTGC | site directed mutagenesis with pTMJ as template | pTMJ-3D |
| ACG-2A-J | ACACCAACTGCCATCTTGGACGGGTATTGTG | site directed mutagenesis with pTMJ as template | pTMJ-2A |
| ACG-1T-J | ACACCAACTGCCATAGTGGACGGGTATTGTG | site directed mutagenesis with pTMJ as template | pTMJ-1T |
| ACG-GUG-J | AGACACCAACTGCCACCGTGGACGGGTATT | site directed mutagenesis with pTMJ as template | pTMJ-GUG |
| ACG-UUG-J | AGACACCAACTGCCAACGTGGACGGGTATT | site directed mutagenesis with pTMJ as template | pTMJ-UUG |
| ACG+4T-J | AGCAGACACCAACTGACATCGTGGACGGG | site directed mutagenesis with pTMJ as template | pTMJ+4T |
| ACG+4A-J | AGCAGACACCAACTGTCATCGTGGACGGG | site directed mutagenesis with pTMJ as template | pTMJ+4A |
| ACG+4C-J | AGCAGACACCAACTGGCATCGTGGACGGG | site directed mutagenesis with pTMJ as template | pTMJ+4C |
| D3-J | GACACCAACTGCCATGGACGGGTATTGTGC | site directed mutagenesis with pTMJ as template | pTMJ-D3 |
| D3-GUG-J | AGACACCAACTGCCACGGACGGGTATTGTGC | site directed mutagenesis with pTMJ-D3 as template | pTMJ-D3-GUG |
| D3-UUG-J | AGACACCAACTGCCAAGGACGGGTATTGTGC | site directed mutagenesis with pTMJ-D3 as template | pTMJ-D3-UUG |
| D4-J | GACACCAACTGCCATGACGGGTATTGTGCG | site directed mutagenesis with pTMJ as template | pTMJ-D4 |
| D4-GUG-J | AGACACCAACTGCCACGACGGGTATTGTGCGC | site directed mutagenesis with pTMJ-D4 as template | pTMJ-D4-GUG |
| D4-UUG-J | AGACACCAACTGCCAAGACGGGTATTGTGCGC | site directed mutagenesis with pTMJ-D4 as template | pTMJ-D4-UUG |
| R-H | CAGACACCAACTGCCATCTTGCCCGCAAATAC | site directed mutagenesis with pTM-H as template | pTMH |
| R-3C-H | CACCAACTGCCATCTGGCCCGCAAATACCGT | site directed mutagenesis with pTMH as template | pTMH-3C |
| R+4T-H | AGCAGACACCAACTGACATCTTGCCCGCAAA | site directed mutagenesis with pTMH as template | pTMH+4T |
| R-N | ACACCAACTGCCATGTTACCCGACAGTACGG | site directed mutagenesis with pTM-N as template | pTMN |
| R-3C-N | CACCAACTGCCATGTGACCCGACAGTACGGA | site directed mutagenesis with pTMN as template | pTMN-3C |
| R+4T-N | AGCAGACACCAACTGACATGTTACCCGACAG | site directed mutagenesis with pTMN as template | pTMN+4T |
| Kozak-R2 | ATAGGTACCCGCGGTCCGGAGAGC | amplification of the partial *bgaH* ORF | **”** |
| 16S rRNA-RT-f | TGCTTTGACGGGCGGTGTGT | amplification of 16S rRNA (Realtime PCR) | **-** |
| 16S rRNA-RT-r | CGGTAGGTCAGTATGCCCCGAATG | amplification of 16S rRNA (Realtime PCR) | **-** |
| *bgaH*-RT-f | TCGGTATGAAAGCGGTTCTGTGC | amplification of *bgaH* (Realtime PCR) | **-** |
| *bgaH*-RT-r | CCACAGTCCTCGCAGTAGCAGGTAA | amplification of *bgaH* (Realtime PCR) | **-** |
